# Eliciting Dose and Safety Outcomes From a Large Dataset of Standardized Multiple Food Challenges

**DOI:** 10.3389/fimmu.2018.02057

**Published:** 2018-09-21

**Authors:** Natasha Purington, R. Sharon Chinthrajah, Andrew Long, Sayantani Sindher, Sandra Andorf, Katherine O'Laughlin, Margaret A. Woch, Alexandra Scheiber, Amal Assa'ad, Jacqueline Pongracic, Jonathan M. Spergel, Jonathan Tam, Stephen Tilles, Julie Wang, Stephen J. Galli, Manisha Desai, Kari C. Nadeau

**Affiliations:** ^1^Sean N. Parker Center for Allergy and Asthma Research, Stanford University School of Medicine, Stanford, CA, United States; ^2^Quantitative Sciences Unit, Stanford University School of Medicine, Stanford, CA, United States; ^3^Department of Pharmacy, Lucile Packard Children's Hospital Stanford, CA, United States; ^4^Division of Allergy and Immunology, Cincinnati Children's Medical Center, Cincinnati, OH, United States; ^5^Division of Allergy and Immunology, The Ann and Robert H. Lurie Children's Hospital of Chicago, Chicago, IL, United States; ^6^Division of Allergy and Immunology, Department of Pediatrics, The Children's Hospital of Philadelphia, Perelman School of Medicine at University of Pennsylvania, Philadelphia, PA, United States; ^7^Division of Clinical Immunology and Allergy, Children's Hospital Los Angeles, Los Angeles, CA, United States; ^8^ASTHMA Inc. Clinical Research Center, Northwest Asthma and Allergy Center, University of Washington, Seattle, WA, United States; ^9^Division of Allergy and Immunology, Department of Pediatrics, Icahn School of Medicine at Mount Sinai, New York, NY, United States; ^10^Department of Pathology, Stanford University School of Medicine, Stanford, CA, United States; ^11^Department of Microbiology and Immunology, Stanford University School of Medicine, Stanford, CA, United States

**Keywords:** oral food challenge, adverse events, dose curves, food allergy, safety outcome

## Abstract

**Background:** Food allergy prevalence has continued to rise over the past decade. While studies have reported threshold doses for multiple foods, large-scale multi-food allergen studies are lacking. Our goal was to identify threshold dose distributions and predictors of severe reactions during blinded oral food challenges (OFCs) in multi-food allergic patients.

**Methods:** A retrospective chart review was performed on all Stanford-initiated clinical protocols involving standardized screening OFCs to any of 11 food allergens at 7 sites. Interval-censoring survival analysis was used to calculate eliciting dose (ED) curves for each food. Changes in severity and ED were also analyzed among participants who had repeated challenges to the same food.

**Results:** Of 428 participants, 410 (96%) had at least one positive challenge (1445 standardized OFCs with 1054 total positive challenges). Participants undergoing peanut challenges had the highest ED_50_ (29.9 mg), while those challenged with egg or pistachio had the lowest (7.07 or 1.7 mg, respectively). The most common adverse event was skin related (54%), followed by gastrointestinal (GI) events (33%). A history of asthma was associated with a significantly higher risk of a severe reaction (hazard ratio [HR]: 2.37, 95% confidence interval [CI]: 1.36, 4.13). Higher values of allergen-specific IgE (sIgE) and sIgE to total IgE ratio (sIgEr) were also associated with higher risk of a severe reaction (1.49 [1.19, 1.85] and 1.84 [1.30, 2.59], respectively). Participants undergoing cashew, peanut, pecan, sesame, and walnut challenges had more severe reactions as ED increased. In participants who underwent repeat challenges, the ED did not change (*p* = 0.66), but reactions were more severe (*p* = 0.02).

**Conclusions:** Participants with a history of asthma, high sIgEr, and/or high values of sIgE were found to be at higher risk for severe reactions during food challenges. These findings may help to optimize food challenge dosing schemes in multi-food allergic, atopic patients, specifically at lower doses where the majority of reactions occur.

**Trials Registration Number:** ClinicalTrials. gov number NCT03539692; https://clinicaltrials.gov/ct2/show/NCT03539692.

## Introduction

The prevalence of food allergies has continued to rise over the past decade and has become a significant health issue (1). Food allergies have become more common, and now affect 6–11% of the population in the United States, Canada, Australia, and Europe (2–8). Among children, 40% are affected by two or more food allergies (9). The diagnosis of food allergies imposes a significant burden on patients and their families and leads to a decreased quality of life due to dietary restrictions, increased anxiety, and social limitations (10). In recent years, in the US, the number of emergency room visits for food-induced anaphylaxis has risen to ~200,000/year and continues to rise (11, 12).

The double-blind placebo-controlled food challenge (DBPCFC) is the gold standard method to diagnose food allergies. Recent studies have focused on comparing the utility of other clinical factors to be able to predict food challenge outcomes (13) and to understand the role of allergen-specific IgE (sIgE) and skin prick tests (SPTs) (14). However, there have been few comparisons of multiple DBPCFCs performed across a large population in which the challenges were done with the same standardized method. In a prior publication from our group (15), we demonstrated the presence of multiple food allergies in many individuals. Our sites perform clinical trials in food allergy and as such, a large number of DBPCFCs are conducted in a medical facility with trained personnel using the same doses and time intervals in a food challenge. Sometimes participants undergo repeat food challenges (without interim intervention) to the same allergen for qualification into clinical trials. Therefore, the objective of this research was to test whether food challenge reactions, if repeated over time, differed by severity, by eliciting dose (ED), or by organ system involvement. This was determined according to the type or dose of food allergen (16, 17). Another objective was to assess whether certain food allergens were associated with a certain type of reaction (i.e. a gastrointestinal (GI) allergic reaction vs. a skin allergic reaction).

## Materials and methods

### Oral food challenges (OFCs)

From September 2010 to March 2016, participants with suspected food allergy were recruited to undergo standardized food challenges to at least 500 mg of cumulative food protein to each of their allergens as part of screening for clinical trial enrollment. The low cutoff of 500 mg of food protein was chosen as these subjects had a high likelihood of exhibiting an allergic reaction. The precise amounts of commercially available, FDA standardized and validated GMP-grade protein were quantified based on protein gels, prepared and weighed out in our GMP facility, and distributed to other sites under a clinical trial agreement that ensured consistency in challenge material from batch to batch and between sites. Patients with a prior history of food-allergy reaction requiring intubation or eliciting hypotension were excluded, while patients with previous reactions to food requiring epinephrine for other severe symptoms were eligible. During the initial screening visit before multiple studies, SPT and IgE testing were performed at the Center for some trials, whereas, for others, results from prior testing at a physician's office were included. SPT consisted of a positive histamine control, a negative saline control (both from Hollister-Stier) and allergen extracts from Greer. SPTs were performed on the volar surface of the forearm or back after application of the respective allergen solution. Mean wheal diameter was measured after 20 min. Allergen-specific IgE levels were measured by ImmunoCAP fluorescence enzyme immunoassay.

One thousand four hundred and forty-five DBPCFCs were performed using standardized methodology according to validated guidelines (18–20). The same DBPCFC methods and doses were used across the Sean N. Parker Center for Allergy and Asthma Research at Stanford University, Cincinnati Children's Medical Center, Robert H. Lurie Children's Hospital of Chicago, Children's Hospital of Philadelphia, Virginia Mason Medical Center, Seattle Children's Hospital, Icahn School of Medicine at Mount Sinai, and Children's Hospital Los Angeles. All personnel were trained using procedures as per the protocol. Each challenge consisted of several escalating doses of the food protein in flour form concealed in an appropriate vehicle, such as applesauce or pudding, ingested by the participant every 15 min as tolerated. Challenges to almond, cashew, egg, hazelnut, milk, peanut, pecan, pistachio, sesame, walnut, and wheat were included in the analyses. Typically challenges started with as small as 1 mg (for pistachio), then 2, 5, 20, 50, 100, 100, 100, 123 (for pistachio), or 124 mg. Patients challenged with pistachio were individuals with a known cashew allergy, and, as such, pistachio challenges were started at 1 mg due to concerns for safety. All allergen doses indicate mg of food protein. Those participants with positive DBPCFCs to placebo (oat) were excluded. A subset of patients performed repeat challenges to the same food in the course of screening for multiple trials. Vital signs and pertinent physical examinations were repeated every 15 min, or more frequently during the challenge, at the discretion of the clinician. Reaction types and severities were determined according to modified Bock criteria (18) and Common Terminology Criteria for Adverse Events (CTCAE v 4.03). Some studies recorded symptoms in CTCAE criteria and some with modified Bock. Our ranking system was based on Bock and the CTCAE was converted to Bock grading by allergists on our team. All objective and subjective symptoms were recorded and ranked against one another in order of severity by onsite physicians based on their clinical judgment. Subjective symptoms included abdominal pain, oropharyngeal itching, nausea, or pruritus. Objective adverse symptoms were regarded as more severe than subjective symptoms of the same grade and this was taken into consideration when ranking symptoms in Table 1. Participants tolerating at least 500 mg cumulative dose during the challenge were considered to be negative responders for the purposes of this analysis. All aspects of the studies from which data was obtained were authorized by the IRB.

**Table 1:** Ranked adverse events by severity.

**Symptom**	**Rank**
Mild pruritus	1
Moderate pruritus	2
Mild nasal itching	3
Moderate nasal itching	4
Severe nasal itching	5
Mild nausea	6
Moderate nausea	7
Severe nausea	8
Mild Ab pain	9
Moderate Ab pain	10
Mild rhinorrhea	11
Mild nasal congestion	12
Moderate rhinorrhea	13
Mild sneezing	14
Moderate nasal congestion	15
Mild rash	16
Mild urticaria	17
Moderate sneezing	18
Mild angioedema	19
Severe rhinorrhea	20
Severe nasal congestion	21
Mild cough	22
Severe sneezing	23
Mild emesis	24
Severe Ab pain	25
Severe pruritus	26
Moderate rash	27
Moderate emesis	28
Moderate angioedema	29
Moderate cough	30
Moderate urticaria	31
severe rash	32
Severe urticaria	33
severe emesis	34
Severe angioedema	35
Severe cough	36
Mild airway obstruction	37
Moderate airway obstruction	38
Severe airway obstruction	39
Mild wheezing	40
Moderate wheezing	41
Severe wheezing	42
Mild cardio	43
Moderate cardio	44
Severe cardio	45

*Higher ranking indicates more severe symptoms*.

### Data management

Any value of sIgE greater than 100 IU/L was truncated to 101 for statistical analysis. Only SPT and/or sIgE that were collected within 12 months of the OFC were included in the analysis. If a subject had more than one value for SPT or sIgE, then the value obtained closest to the challenge was used (14). Negative control SPTs were subtracted from the raw food SPTs prior to analysis. If the newly derived SPT was negative, it was set to zero. Any SPT that was collected after the food challenge or collected more than 12 months before the challenge was excluded. If a subject had more than one value for either SPT or sIgE, then the value obtained most recently was used.

In an effort to standardize OFCs across studies, challenges that were considered positive in their original studies based on thresholds higher than 500 mg but had cumulative tolerated doses (CTDs) of 500 mg or higher were re-classified as having negative challenges with no eliciting dose (ED) to a cumulative of 500 mg of protein. Subjects who had unknown or non-reported ethnicity were coded as missing ethnicity. Subjects with race of Native Hawaiian, other, or not reported were coded as other. Only positive challenge data were analyzed.

### Statistical analysis

To determine how often participants were allergic to multiple foods, pairwise comparisons of all major foods were conducted. The Jaccard similarity coefficient was implemented, accounting for the different number of participants allergic to each food (21). A detailed description of this method and its implementation in food studies has been previously published (22). Only participants who conducted food challenges for multi-food studies were included in this analysis.

To determine ED curves for each challenge food, data were analyzed using interval-censoring survival analysis fitted to three different probability distributions (Log-Normal, Log-Logistic, and Weibull) to estimate the ED for 5, 10, and 50% of patients (23).The three distributions were compared for each food, and the one with the lowest Akaike information criteria (AIC) was chosen. Interval-censoring analysis uses the lowest- and no-observed adverse effect levels (LOAELs and NOAELs) based on challenge information (23). If a participant reacted at the first challenge dose, the NOAEL was set to zero and the LOAEL was set to the first challenge dose. Turnbill intervals were implemented due to overlapping dose steps from various studies. The estimated ED and 95% confidence intervals were reported at each ED level. SAS's PROC LIFEREG was used to implement the analysis (24).

Multiple symptoms could have been reported during each challenge based on participant symptoms. Based on clinical reasoning, all 45 possible symptoms (3 grades for each of the 15 symptoms) were ranked in order of severity (Table 1). This list was then used to select the most “severe” symptom reported from each challenge. Therefore, only the most severe symptom reported [grade and SOC (system organ class)] was analyzed per challenge. Frailty models were fit to “time” (i.e., eliciting dose) until the most severe symptom as a function of each clinical and demographic feature. An event was defined by whether or not the most severe symptom observed was a Bock grade 3. For each model, each participant contributed multiple observations corresponding to the number of food challenges. Due to possible correlations within participant or within food, random effects for participant and food were included in each model. Hazard ratios and 95% CIs were reported. Further, the correlation between ED and the severity ranking was measured by challenge food using the Spearman rank correlation test.

A subset of participants was challenged to the same food twice. The Kruskal-Wallis rank sum test was used to test whether ED changed from the first to second challenge. Spearman's rank order correlation was used to assess the association between change in ED and number of months between repeat challenges. These two tests were also used to assess changes in the symptom severity ranking. Lastly, Spearman's rank order correlation was also used to determine if change in ED was associated with change in symptom rank. *P*-values were reported.

All analyses were conducted at the 0.05 alpha level. No adjustments for multiple comparisons were made. Analyses were conducted using R v.3.4.3 (25) and SAS Software (24). Data are available and can be found on a secure REDcap database that is part 11 compliant.

## Results

### Baseline demographics

Age of participants (*n* = 410) ranged from 1 to 52, with a median age of 9 years old, and the cohort was comprised of mostly non-Hispanic (97%), Caucasian (62%), and males (61%). The majority of participants also had an atopic history, including asthma (62%), allergic rhinitis (77%), and atopic dermatitis (74%). The average number of doctor-diagnosed food allergies was 5, with only 2% of the cohort being mono-food allergic. The median total IgE (tIgE) was 499 kU/L (Table 2).

**Table 2:** Baseline demographics.

Characteristic^*^	**Total (*n* = 410)**
Age in years, median (range)	9 (1–52)
Male	250 (61%)
Non-hispanic	390 (97%)
**RACE**	
Caucasian	250 (62%)
Black	6 (1%)
Asian	106 (26%)
Multiracial	37 (9%)
Other	5 (1%)
**ATOPIC HISTORY**	
Asthma	232 (62%)
Allergic rhinitis	284 (77%)
Atopic dermatitis	272 (74%)
Number of food allergens, median (range)	5 (1–16)
Mono-food allergic	8 (2%)
Total IgE (IU/L), median (range)	498.5 (18–3366)

* *Count and percent of total subjects unless otherwise noted*.

### Challenge overview

**Figure 1:**
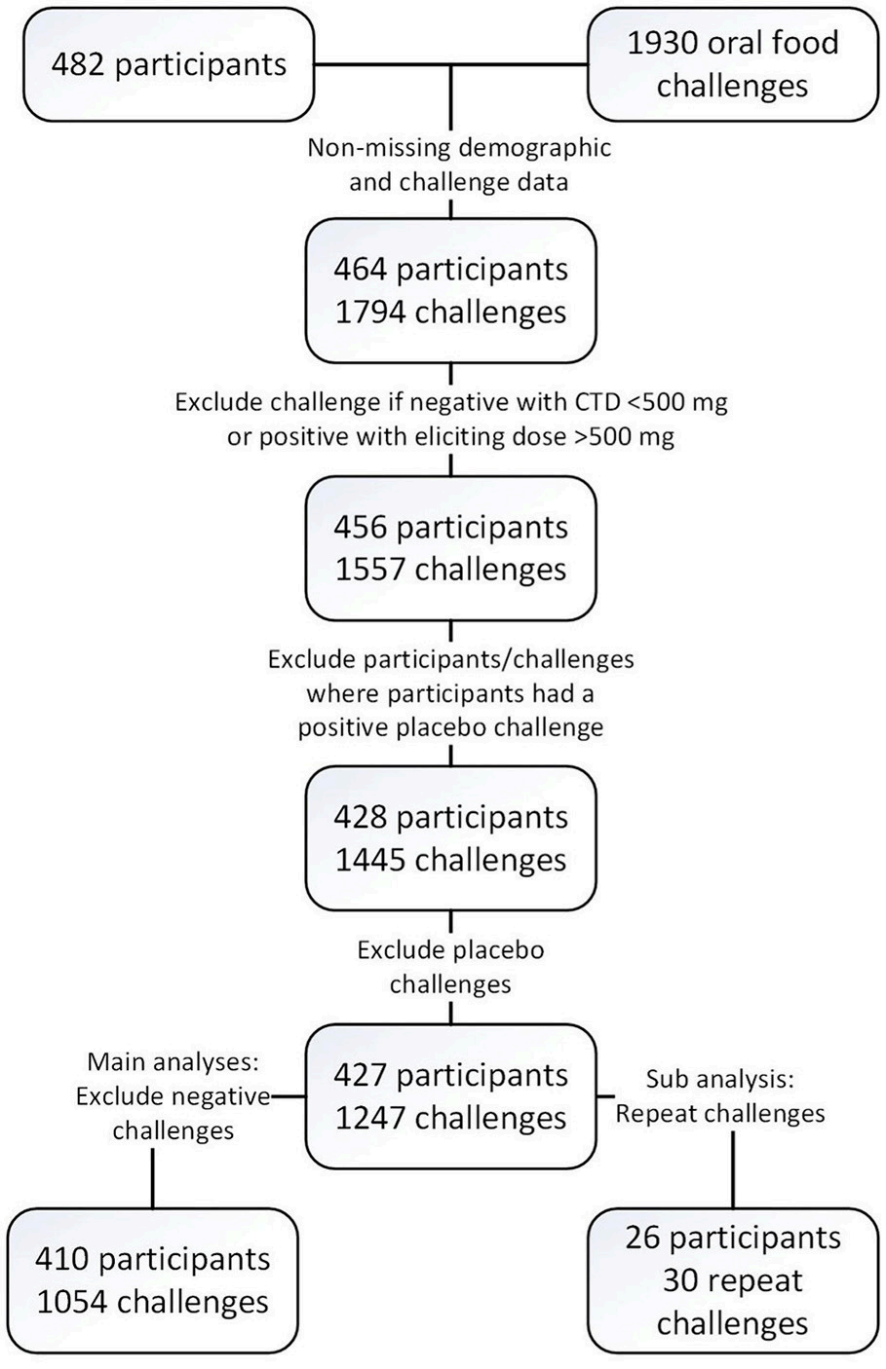
Consort diagram.

Four hundred and twenty-seven participants across multiple studies contributed 1,445 baseline challenges to the database (Figure 1 and Table 3) of which 410 had 1,054 positive challenge outcomes. The most common positive challenge was for peanut (*n* = 347) followed by cashew (*n* = 151) and walnut (*n* = 121; Table 3). Seventy-seven percent of participants had a peanut allergy.

**Table 3:** Eliciting dose (ED) thresholds by food.

**Challenge Food**	**N**	**Number of subjects (% of total)**	**Eliciting dose (mg) median (range)**	**Eliciting dose curves (ED) (mg) (95% CI)**		
				**ED_5_**	**ED_10_**	**ED_50_**
Almond^✤^	30	29 (7)	25.0 (5–500)	0.86 (0, 1.92)	1.73 (0, 3.60)	20.77 (5.76, 35.78)
Cashew	151	150 (35)	25.0 (0.1–500)	0.07 (0, 0.13)	0.25 (0.05, 0.46)	8.78 (5.40, 12.16)
Egg	63	60 (14)	8.1 (0.1–500)	0.04 (0, 0.12)	0.18 (0, 0.42)	7.07 (2.61, 11.54)
Hazelnut	68	65 (15)	25.0 (1.6–500)	0.07 (0, 0.17)	0.29 (0, 0.68)	14.38 (5.36, 23.39)
Milk	67	66 (15)	32.7 (1.7–500)	0.21 (0, 0.49)	0.74 (0, 1.55)	20.41 (9.73, 31.09)
Peanut	347	330 (77)	75.0 (0.1–500)	0.49 (0.24, 0.73)	1.52 (0.89, 2.15)	29.90 (23.81, 35.98)
Pecan^✤^	88	88 (21)	25.0 (1.7–500)	0.38 (0.04, 0.71)	0.79 (0.19, 1.39)	10.68 (5.71, 15.64)
Pistachio	60	59 (14)	5.0 (5–275)	0 (0, 0.1)	0.01 (0, 0.04)	1.71 (0, 3.61)
Sesame	30	30 (7)	25.0 (5–500)	0.26 (0, 0.75)	0.88 (0, 2.24)	21.19 (5.28, 37.10)
Walnut	121	120 (28)	25.0 (1.7–500)	0.15 (0, 0.31)	0.56 (0.07, 1.05)	18.01 (10.54, 25.47)
Wheat	13	13 (3)	32.7 (5–500)	0.03 (0, 0.17)	0.16 (0, 0.75)	12.64 (0, 33.20)

*All models fit to Weibull distribution unless otherwise noted by^✤^ (Log-normal)*.

**Figure 2:**
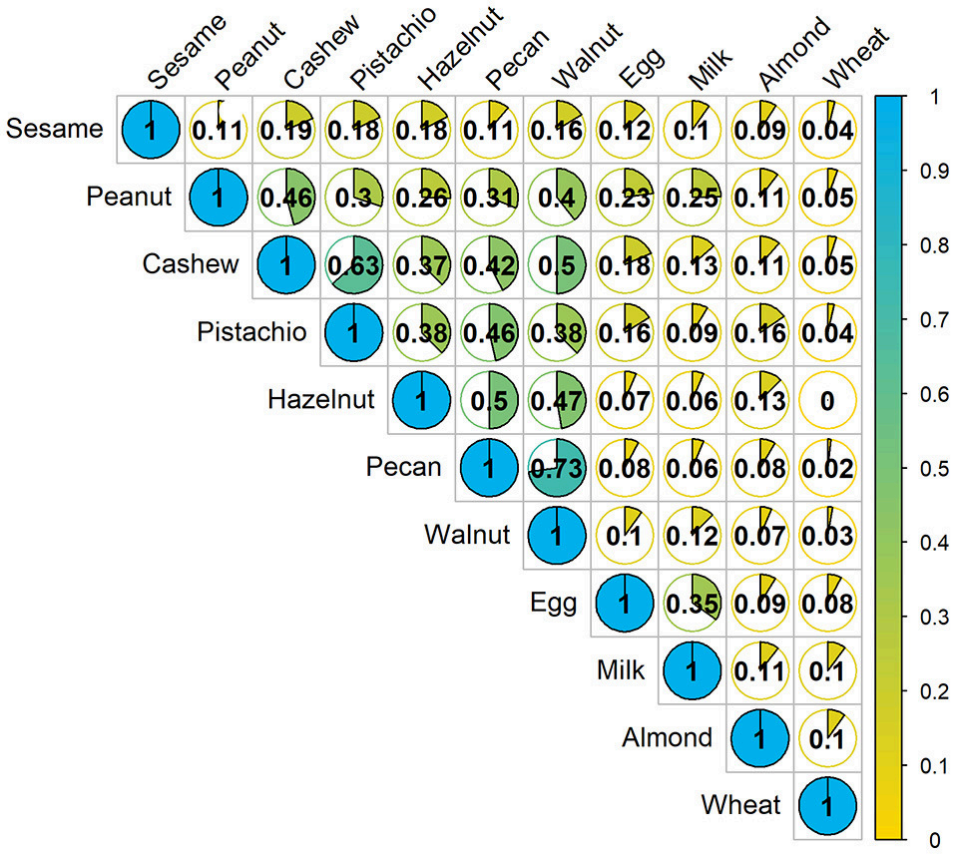
Concurrent occurrences of food allergy based on food challenge outcomes: The fraction in each cell represents the Jaccard similarity coefficient, which is the amount of co-allergy accounting for the number of positive challenges for each allergen separately. Higher values indicate more similarity between the two allergens. Denominator only includes participants who were screened for multi-food allergy studies.

A Jaccard analysis assessing the similarity of co-allergy among the foods which were challenged in our cohort is illustrated in Figure 2. A higher similarity index corresponds to a higher degree of overlap of results obtained between two foods. Overall, higher similarity was observed within peanut and tree nut allergies compared to milk, egg, wheat or sesame. Allergies to pecan and walnut were 73% similar, followed by cashew and pistachio, which were 63% similar.

### Eliciting dose

**Figure 3:**
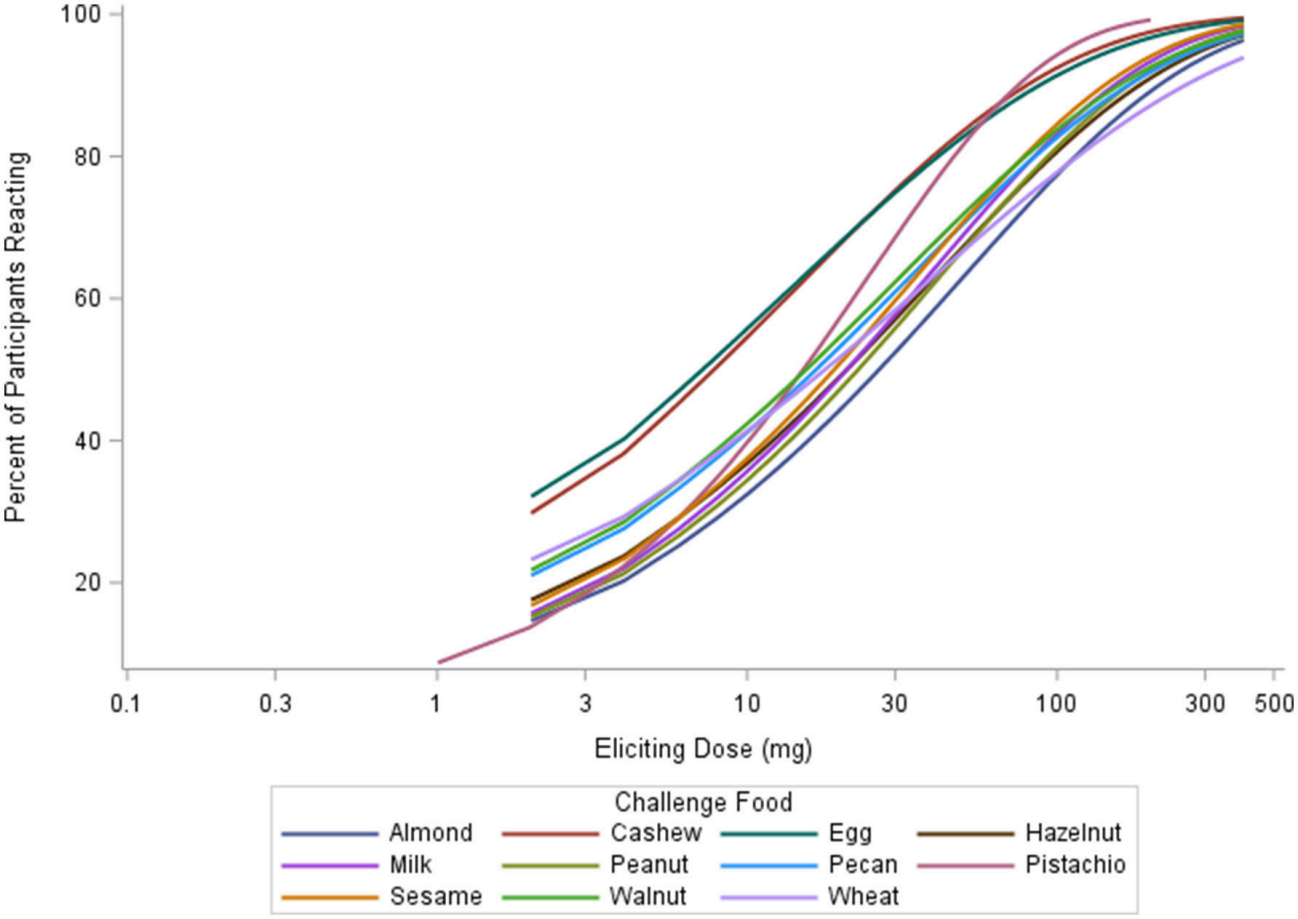
Eliciting dose (ED) thresholds by allergen.

The median ED was <35 mg of food protein for all foods, except for peanut, with the highest median ED at 75 mg, and pistachio, having the lowest at 5 mg (Table 3). Participants undergoing peanut challenges had the highest ED_50_ dose (i.e., the dose which elicits a reaction in 50% of subjects in those that ultimately react) of all foods (29.9 mg), followed by sesame (21.2 mg) and almond (20.7 mg). Pistachio had the lowest dose to elicit a reaction in 50% of subjects at 1.7 mg, however, only the participants with a positive reaction to cashew were challenged with pistachio. Participants challenged with egg had the second lowest ED_50_ dose (7.07 mg). Across each of the three ED thresholds, almond and peanut consistently had the highest dose values. A higher percentage of participants challenged with egg and cashew reacted at lower EDs compared to other foods (Figure 3). Participants undergoing pistachio challenges had the largest increase in reactions over EDs than any other food, while participants with wheat had the lowest increase in percentage of participants reacting.

### Adverse events

**Figure 4:**
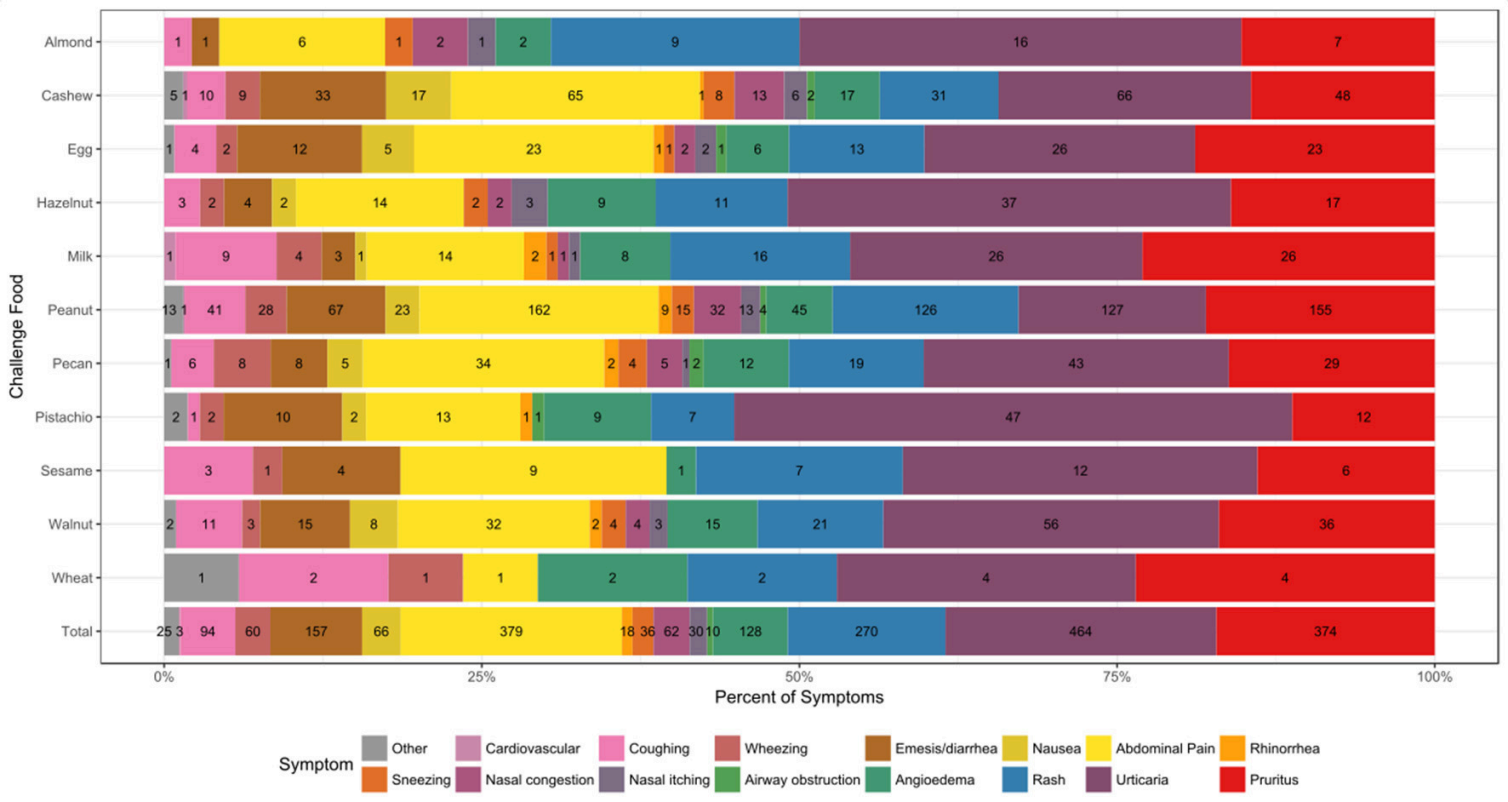
Symptom type by allergen. Reaction counts are reported within the figure.

A total of 2014 adverse events occurred during the 1,054 positive challenges (Table 4). The majority of adverse events occurred during peanut challenges (*n* = 795) followed by cashew (*n* = 312), which were also the most frequent challenges conducted. Within each food, adverse events related to skin were the most prevalent (54%), followed by GI events (33%). More specifically, urticaria and pruritus were the most common skin reactions, while abdominal pain was the most common GI reaction (Figure 4). The distribution of symptom type was similar across foods.

**Table 4:** Adverse events by allergen and organ system.

	**Number of AEs (% Total)**				**Total**
**Allergen**	**Gastrointestinal**	**Respiratory**	**Skin**	**Other**	
Almond	9 (20.5)	3 (6.8)	32 (72.7)	0 (0.0)	44
Cashew	116 (37.2)	40 (12.8)	150 (48.1)	6 (1.9)	312
Egg	42 (36.8)	14 (12.3)	57 (50.0)	1 (0.9)	114
Hazelnut	22 (23.2)	10 (10.5)	63 (66.3)	0 (0.0)	95
Milk	23 (21.1)	14 (12.8)	71 (65.1)	1 (0.9)	109
Peanut	292 (36.7)	108 (13.6)	389 (48.9)	6 (0.8)	795
Pecan	49 (29.7)	20 (12.1)	95 (57.6)	1 (0.6)	165
Pistachio	26 (28.0)	6 (6.5)	61 (65.6)	0 (0.0)	93
Sesame	18 (39.1)	3 (6.5)	25 (54.3)	0 (0.0)	46
Walnut	61 (31.3)	23 (11.8)	110 (56.4)	1 (0.5)	195
Wheat	1 (7.1)	2 (14.3)	10 (71.4)	1 (7.1)	14
Total	666 (33.1)	247 (12.3)	1084 (53.8)	17 (0.8)	2014

Table 1 lists the ordered rank of the potential adverse events that could occur during each participant's challenge, with lower ranked adverse events corresponding to more concerning symptoms. For example, severe cardiac symptoms, with a severity grade of 3, was ranked as number 45, compared to pruritus, with a severity grade of grade 1, which was ranked as number 1. Among the lower ranked adverse events (based on modified Bock criteria) (18), 673 (74%) were graded as mild, 134 (15%) as moderate, and 98 (11%) as severe (data not shown).

**Figure 5:**
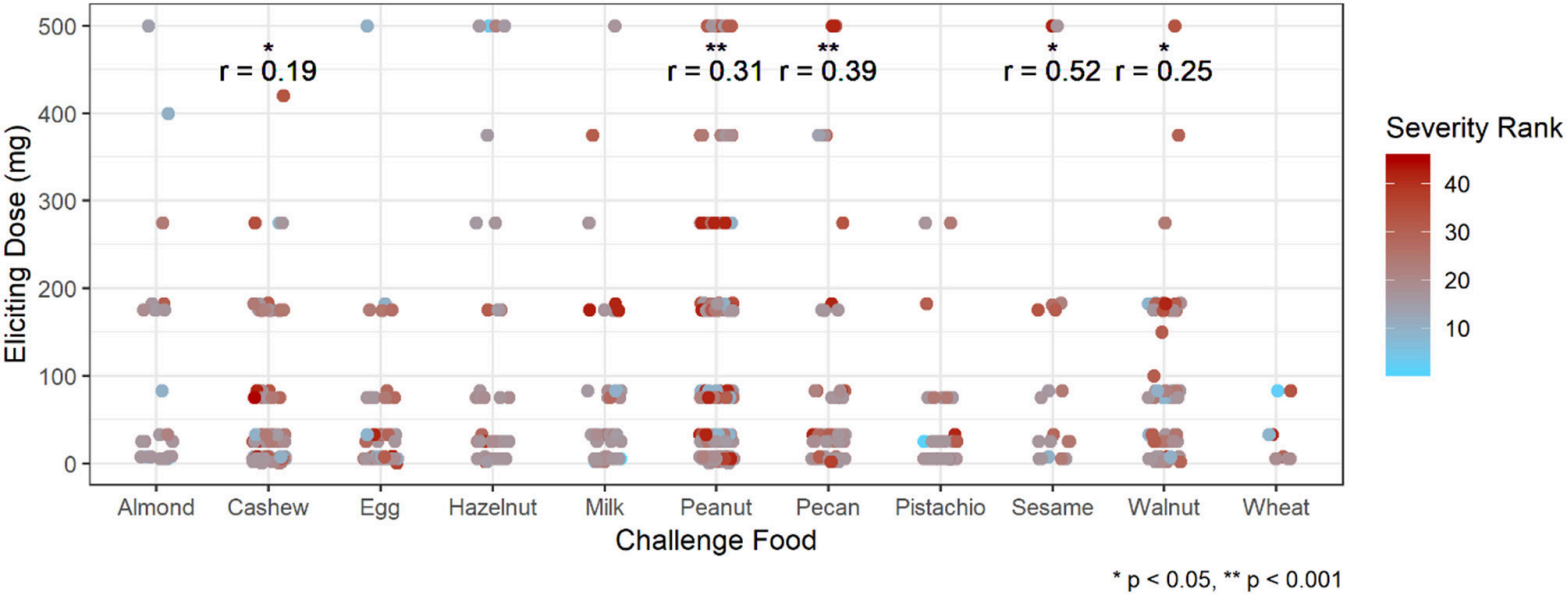
Correlation of eliciting dose and adverse event severity ranking by challenge food. ^*^p < 0.05, ^**^p < 0.001 by Spearman rank correlation test. Red ranking corresponds to more severe symptoms, while blue corresponds to more mild symptoms.

Participants with a history of asthma were more than twice as likely to have their most severe AE be a Bock grade of 3 at any point in their challenge compared to those without a history of asthma (hazard ratio [HR]: 2.37, 95% confidence interval [CI]: 1.36, 4.13; Table 5). Higher values of sIgE and sIgEr were significantly associated with higher risk of experiencing a severe reaction [HR: 1.49 [1.19, 1.85] and 1.84 [1.30, 2.59], respectively]. Participants who were challenged with cashew, peanut, pecan, sesame, and walnut had a higher severity ranking that was significantly associated with higher ED and, as ED increased, so did the severity (Figure 5).

**Table 5:** Univariate associations of severity.

**Characteristic**	**Not Severe**	**Severe**	**Hazard ratio (95% CI)**	**Challenges included**
Female	40%	39%	0.96 (0.6, 1.53)	905
Hispanic	98%	99%	1.11 (0.14, 8.74)	887
Race (ref = Caucasian)				895
Black	1%	1%	0.93 (0.11, 7.88)	
Asian	29%	37%	1.56^*^ (0.95, 2.59)	
Multiracial	11%	5%	0.62 (0.23, 1.69)	
**ATOPIC HISTORY**				
Asthma	60%	76%	2.37^**^ (1.36, 4.13)	825
Allergic rhinitis	77%	82%	1.1 (0.59, 2.04)	812
Atopic dermatitis	77%	75%	1.05 (0.59, 1.86)	813
Age	8	8	0.99 (0.96, 1.03)	905
FEV_1_	99	99	1 (0.98, 1.03)	494
FEV_1_/FVC	0.85	0.86	4.23 (0.04, 457.57)	492
Mono-Allergic	2%	2%	0.51 (0.09, 3.02)	905
Number of diagnosed food allergies	6	5	1 (0.93, 1.09)	905
sIgE (log-scale)	17	43	1.49^***^ (1.19, 1.85)	575
tIgE (log-scale)	439	583	1.2 (0.81, 1.78)	385
sIgEr (log-scale)	0.04	0.06	1.84^***^ (1.3, 2.59)	385
SPT	12	13.5	1.04^*^ (1, 1.08)	600

*Each column corresponds to a single frailty model. SPT, skin prick test; sIgE, allergen-specific Immunoglobulin E; sIgEr, ratio of sIgE to total IgE (tIgE). Values in the “Not Severe” and “Severe” columns are the percentages, means, and medians for each characteristic on the raw scale. Median values are presented for age and each biomarker*.

* p < 0.10;

** p < 0.05;

*** *p < 0.01*.

### Repeat challenges

**Figure 6:**
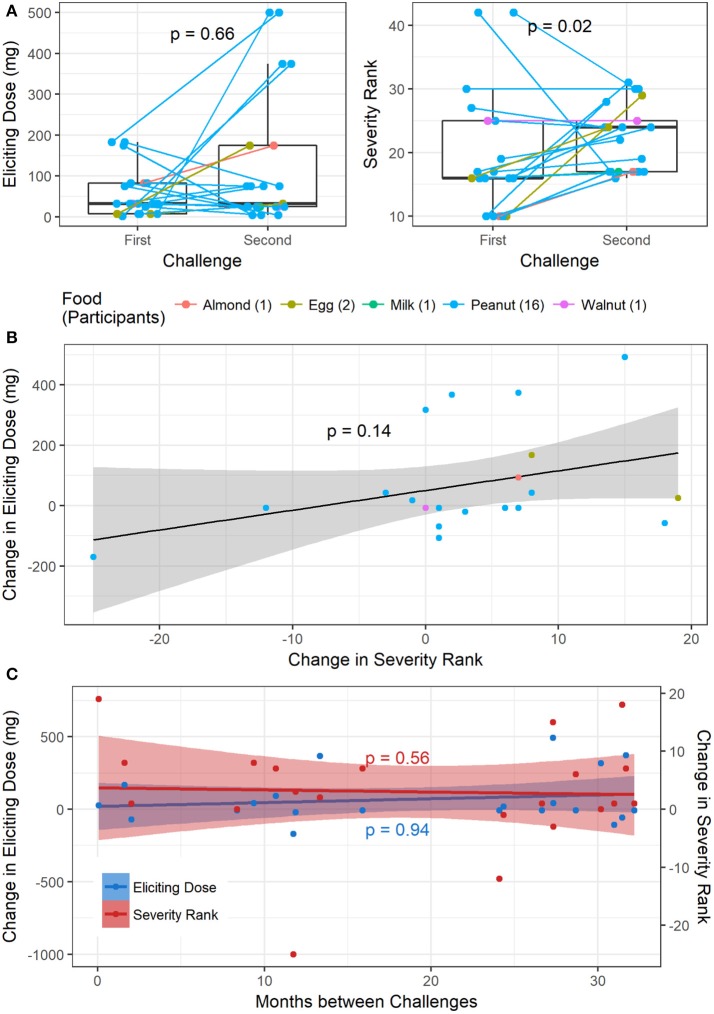
Repeat challenges: **(A)** boxplot of change in ED and severity ranking from first to second challenge. **(B)** Association between change in ED and change in severity ranking. **(C)** Association between change in ED and time between challenges, and change in severity and time between challenges.

Of the 1445 total challenges (positive and negative), 30 were repeated by 26 participants. Only one participant had two repeat challenges to the same allergen (peanut), while all others only repeated a challenge to the same food once. Out of the 1054 positive baseline challenges, 21 were repeats with positive challenge outcomes, corresponding to 18 participants. Sixteen repeat challenges were to peanut, two to egg, and one each to almond, milk, and walnut (Figure 6). One participant had a repeat negative challenge to peanut and another had a repeat negative challenge to almond. The delta change in severity ranking from first to second challenge was significantly different from zero (*p* = 0.04; Wilcoxon signed rank test). Additionally, the median time between repeat challenges was 735 days (range 2–982). While there was no difference in ED from the first to second challenge (*p* = 0.66), the severity rank significantly increased in the second challenge, corresponding to more severe symptoms experienced (*p* = 0.02, Figure 6A). By contrast, there was no significant association between change in ED and change in severity rank from the first to second challenge (*p* = 0.14, Figure 6B). Change in either ED or severity rank was not associated with time between repeat challenges (*p* = 0.94 and *p* = 0.56, respectively, Figure 6C).

## Discussion

The diagnosis of food allergy is highly complex (20, 26). Currently, SPT and sIgE are commonly used; however, these tests have a high false-positive rate, particularly in children, and lack specificity. Individuals who have a positive test but who do not have an allergic reaction to the allergen on ingestion are said to be sensitized to the allergen. Research on more reliable tests for diagnosing allergy such as the Basophil Activation Test (BAT), CRD, sIgE, IgG4, and total IgE (27) is ongoing. Currently, the gold standard for confirming food allergy (rather than food sensitization) is the DBPCFC (20, 26). However, there are several drawbacks in performing DBPCFCs. Presently, standardized dosing strategies for DBPCFCs are not widely practiced, and the optimal dosing schemes across allergens are unknown. DBPCFCs require multiple days of challenges which can significantly increase the cost. The most significant limitation is that food challenges carry the risk of potentially inducing severe anaphylaxis, which may require hospitalization or care in the intensive care unit (28), therefore DBPCFCs are typically performed under clinical supervision by trained staff who are able to recognize and treat any severe food reaction.

Our data show that the ED_50_ across all allergens is below 30 mg of protein; therefore safety in challenges may be increased by including additional steps at lower doses of the challenge. Compared to previously published thresholds by Blom et al. for cashew, egg, peanut, milk, and hazelnut (23), our findings of ED_5_, ED_10_, and ED_50_ were lower. One potential reason for this might be that the majority of our cohort was multi-food allergic (98%), and highly atopic with over 50% of the cohort with concurrent asthma, allergic rhinitis, and or atopic dermatitis. Additionally, the majority of our challenges had a dosing interval of 15 vs. 30 min reported by Blom et al. Participants undergoing peanut challenges had the highest ED_50_ dose (29.9 mg). Although pistachio had the lowest ED_50_ of 1.7 mg, it represented a small group of participants who had a previous reaction to a cashew challenge. The challenge of such subjects therefore was initiated at a lower dose (of 1 mg) due to safety concerns. Few studies have evaluated prognostic indicators for predicting OFC outcomes (29) and this is an area of ongoing research. In this study we attempted to identify potential prognostic indicators that may be associated with outcomes during OFC to a variety of foods, which could aid in risk stratification for allergists who may be considering a challenge. Our data suggest that food challenges with peanut, sesame, cashew, egg and walnut were more likely to be associated with GI-related symptoms, whereas hazelnut and milk were more likely to be associated with hives. The severity of the reacting symptom is also of concern when conducting a food challenge. Similar to what we and others have shown, a concomitant history of asthma increases the risk of having a severe reaction (29, 30). Not surprisingly, elevated specific IgEs and specific to total IgE ratios were associated with more severe symptoms. However, a severe reaction is possible even at low sIgE values (31). Often, the DBPCFCs conducted for inclusion of clinical trials have more stringent stopping rules and it is felt that more severe symptoms are elicited because of a higher ingested cumulative protein dose. When we assessed the severity of symptoms across doses, we found that severe symptoms were indeed modestly correlated with increasing doses for particular allergens (cashew, peanut, pecan, sesame, and walnut challenges). Perhaps we did not see this for all allergens due to insufficient sample size for those allergens.

In our data set, we also had the unique opportunity to assess ED and the severity of adverse events across repeat food challenges in a small subset of participants. We found that individuals had similar eliciting doses on the first and second challenge, with increasing severity on repeat challenges but with no association with time between challenges, which is consistent with prior findings of repeat challenges (32, 33). However, these results should be interpreted with caution as it is based on a small sample size, limited to 40 repeat challenges, constituting <4% of the total challenges in this cohort. Additionally, the analysis was not adjusted for allergen. Larger cohorts are needed to validate these preliminary findings. CRD was not done and this is a weakness of the paper and will be done in the future.

As food challenges and oral immunotherapy become more popular in outpatient clinics, our findings could provide guidance and better insight into what to expect in performing food challenges in the outpatient clinic setting.

## Ethics statement

This study was carried out in accordance with the recommendations of ICH/GCP/CFR guidelines by the Stanford IRB with written informed consent from all subjects. All subjects gave written informed consent in accordance with the Declaration of Helsinki. The protocol was approved by the Stanford IRB.

## Author contributions

Study was designed by RC, AL, AS, SS, and KN. Study was conducted by RC, AL, AS, SS, AA, JP, JS, JT, ST, JW, and KN. Data analysis was conducted by NP, SA, MD, KN, KL, and MW. Manuscript was written by RC, NP, SA, AL, AS, SS, SG, MD, and KN.

### Conflict of interest statement

The authors declare that the research was conducted in the absence of any commercial or financial relationships that could be construed as a potential conflict of interest.
